# A Theory of Elastic/Plastic Plane Strain Pure Bending of FGM Sheets at Large Strain

**DOI:** 10.3390/ma12030456

**Published:** 2019-02-01

**Authors:** Sergey Alexandrov, Yun-Che Wang, Lihui Lang

**Affiliations:** 1 School of Mechanical Engineering and Automation, Beihang University, No. 37 Xueyuan Road, Beijing 100191, China; sergei_alexandrov@spartak.ru (S.A.); lang@buaa.edu.cn (L.L.); 2 Ishlinsky Institute for Problems in Mechanics, 101-1 Prospect Vernadskogo, 119526 Moscow, Russia; 3 Department of Civil Engineering, National Cheng Kung University, 1 University Road, Tainan 70101, Taiwan

**Keywords:** functionally graded materials, elastoplastic analysis, pure bending, residual stress, large strain

## Abstract

An efficient analytical/numerical method has been developed and programmed to predict the distribution of residual stresses and springback in plane strain pure bending of functionally graded sheets at large strain, followed by unloading. The solution is facilitated by using a Lagrangian coordinate system. The study is concentrated on a power law through thickness distribution of material properties. However, the general method can be used in conjunction with any other through thickness distributions assuming that plastic yielding initiates at one of the surfaces of the sheet. Effects of material properties on the distribution of residual stresses are investigated.

## 1. Introduction

Structures made of functionally graded materials (FGM) are advantageous for many applications. A difficulty with theoretical analysis and design is that structures made of FGM are classified by a much greater number of parameters than similar structures made of homogeneous materials. For this reason, it is desirable to perform parametric studies by analytic or semi-analytic methods as much as possible. A review of results related to the analysis of FGM and published before 2007 is presented in [1]. This review focuses on structures with through-thickness variation of material properties. Analytic solutions derived in [1,2,3,4,5] belong to this class of FGM as well. In [2,3,4], elastic and elastic/plastic spherical vessels subjected to various loading conditions are considered. Thermo-elastic simply supported and clamped circular plates are studied in [5]. Many analytic and semi-analytic solutions are available for FGM discs and cylinders assuming that material properties vary in the radial direction but are independent of the circumferential and axial directions. Purely elastic solutions for a hollow disc or cylinder subjected to internal or/and external pressure are derived in [6,7,8]. An axisymmetric thermo-elastic solution for a hollow cylinder subjected quite a general system of thermo-mechanical loading is presented in [9]. It is assumed that the temperature varies along the radial coordinate. A plane strain analytic elastic/plastic solution for pressurized tubes is found in [10]. The solution is based on the Tresca yield criterion. Many solutions are proposed for functionally graded solid and hollow rotating discs. Purely elastic solutions for solid discs of constant thickness are given in [11,12], a purely elastic solution for a hollow disc of variable thickness in [13], a purely elastic solution for hollow polar orthotropic discs in [14], and a solution for hollow cylinders using the theory of electrothermoelasticity in [15]. An elastic perfectly plastic stress solution for hollow discs is derived in [16] using the von Mises yield criterion.

All of the aforementioned solutions deal with infinitesimal strain. A distinguished feature of the solution provided in the present paper is that strains are large. The process considered is pure bending of a FGM sheet under plane strain conditions. A review on bending of functionally graded sheets and beams at infinitesimal strains is given in [17]. The present solution is based on the approach proposed in [18]. It is shown in this paper that the use of Lagrangian coordinates facilitates the solution. Moreover, the equations describing kinematics can be solved independently of stress equations in the case of isotropic incompressible material. This is an advantage as compared to the classic approach developed in [19] where the stress equations are solved first. The classic approach is restricted to perfectly plastic materials, whereas the mapping in Equation (1) is valid for a large class of constitutive equations. The approach proposed in [18] has already been successfully extended to more general constitutive equations in [20,21,22,23]. It is shown in the present paper that the approach is also efficient for FGM sheets. It is worth noting that a rigid plastic solution for pure bending of laminated sheets (such sheets can also be referred to as functionally graded sheets) at large strain is given in [24].

## 2. Basic Equations

**Figure 1:**
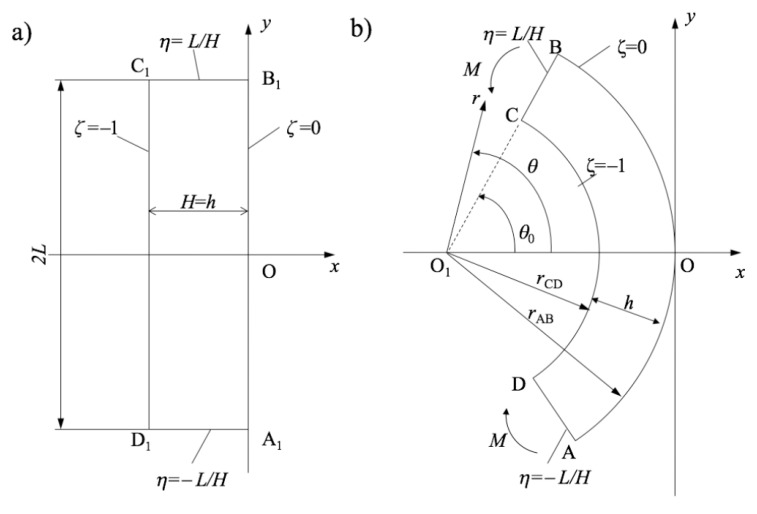
Geometric configuration of the bending problem: (**a**) before deformation; and (**b**) after deformation.

The process of plane strain pure bending is illustrated in Figure 1. The approach proposed in [18] for solving the corresponding boundary value problem is based on the following transformation equations:

(1) $$\begin{array}{c} \frac{x}{H}=\sqrt{\frac{\zeta }{a}+\frac{s}{a^{2}}}\cos\left(2a\eta \right)-\frac{\sqrt{s}}{a},\;\frac{y}{H}=\sqrt{\frac{\zeta }{a}+\frac{s}{a^{2}}}\sin\left(2a\eta \right). \end{array}$$

where (x,y) is an Eulerian–Cartesian coordinate system and (ζ,η) is a Lagrangian coordinate system. Without loss of generality, it is possible to assume that the origin of the Cartesian coordinate system is located at the intersection of the axis of symmetry of the process and the outer surface AB and that the x-axis coincides with the axis of symmetry. The Lagrangian coordinate system is chosen such that

(2) $$\begin{array}{c} \zeta =x/H\;\mathrm{and}\;\eta =y/H \end{array}$$

at the initial instant where *H* is the initial thickness of the sheet. It is evident from these relations and the geometry in Figure 1 that ζ=0 on AB, ζ=−1 on CD, η=L/H on CB and η=−L/H on AD throughout the process of deformation. Here, *L* is the initial width of the sheet. In Equation (1), *a* is a time-like variable. In particular, a=0 at the initial instant. In Equation (1), *s* is a function of *a*. This function should be found from the stress solution and therefore depends on constitutive equations. The condition in Equation (2) is satisfied if

(3) $$\begin{array}{c} s=\frac{1}{4} \end{array}$$

at a=0. It is possible to verify by inspection that the mapping in Equation (1) satisfies the equation of incompressibility. Moreover, this mapping transforms initially straight lines $A_{1}B_{1}$ and $C_{1}D_{1}$ into circular arcs AB and CD and initially straight lines $C_{1}B_{1}$ and $A_{1}D_{1}$ into circular arcs CB and AD after any amount of deformation (Figure 1). Furthermore, coordinate curves of the Lagrangian coordinate system coincide with trajectories of the principal strain rates and, for coaxial models, with trajectories of the principal stresses. Thus, the shear stress vanishes in the Lagrangian coordinates. In particular, the contour ABCD is free of shear stresses. Let $\sigma_{\zeta }$ and $\sigma_{\eta }$ be the physical stress components referred to the Lagrangian coordinates. The stress solution should satisfy the boundary conditions

(4) $$\begin{array}{c} \sigma_{\zeta }=0 \end{array}$$

for ζ=−1 and ζ=0. The only non-trivial equilibrium equation in the Lagrangian coordinates has been derived in [18] as

(5) $$\begin{array}{c} \frac{\partial \sigma_{\zeta }}{\partial \zeta }+\frac{a\left(\sigma_{\zeta }-\sigma_{\eta }\right)}{2\left(\zeta a+s\right)}=0. \end{array}$$

The initial plane strain yield criterion of the functionally graded sheet is supposed to be

(6) $$\begin{array}{c} \left|\sigma_{\zeta }-\sigma_{\eta }\right|=\frac{2}{\sqrt{3}}\sigma_{0}\beta \left(\frac{x}{H}\right). \end{array}$$

where $\sigma_{0}$ is a material constant and β(x/H) is an arbitrary function of its argument. It is assumed that material properties are not affected by plastic deformation. Therefore, Equation (6) can be rewritten in the form

(7) $$\begin{array}{c} \left|\sigma_{\zeta }-\sigma_{\eta }\right|=\frac{2}{\sqrt{3}}\sigma_{0}\beta \left(\zeta \right). \end{array}$$

In this case, the yield locus is invariant along the motion. The importance of this property of material models has been emphasized in [25]. Let $\tau_{\zeta }$ and $\tau_{\eta }$ be the deviatoric portions of $\sigma_{\zeta }$ and $\sigma_{\eta }$, respectively. Since the material is incompressible, $\tau_{\zeta }+\tau_{\eta }=0$ under plane strain conditions. Then, the yield criterion in Equation (7) is equivalent to

(8) $$\begin{array}{c} \left|\tau_{\zeta }\right|=\left|\tau_{\eta }\right|=\frac{\sigma_{0}\beta \left(\zeta \right)}{\sqrt{3}}. \end{array}$$

Hooke’s law generalized on functionally graded materials reads

(9) $$\begin{array}{c} \tau_{\zeta }=2G_{0}g\left(\zeta \right)\varepsilon_{\zeta }^{e},\;\tau_{\eta }=2G_{0}g\left(\zeta \right)\varepsilon_{\eta }^{e}. \end{array}$$

It has been taken into account here that Poisson’s ratio is equal to 1/2 for incompressible materials. In addition, $\varepsilon_{\zeta }^{e}$ and $\varepsilon_{\eta }^{e}$ are the total strain components in elastic regions and the elastic portions of the total strain components in plastic regions referred to the Lagrangian coordinate system, $G_{0}$ is a material constant and g(ζ) is an arbitrary function of its argument.

Geometric parameters shown in Figure 1 depend on *a* and are expressed as [18]

(10) $$\begin{array}{c} \frac{r_{\mathrm{AB}}}{H}=\frac{\sqrt{s}}{a},\;\frac{r_{\mathrm{CD}}}{H}=\frac{\sqrt{s-a}}{a},\;\theta_{0}=\frac{2aL}{H},\;\frac{h}{H}=\frac{\sqrt{s}-\sqrt{s-a}}{a}. \end{array}$$

Once *s* has been found as a function of *a*, these parameters are immediate from Equation (10).

## 3. Stress Solution at Loading

**Figure 2:**
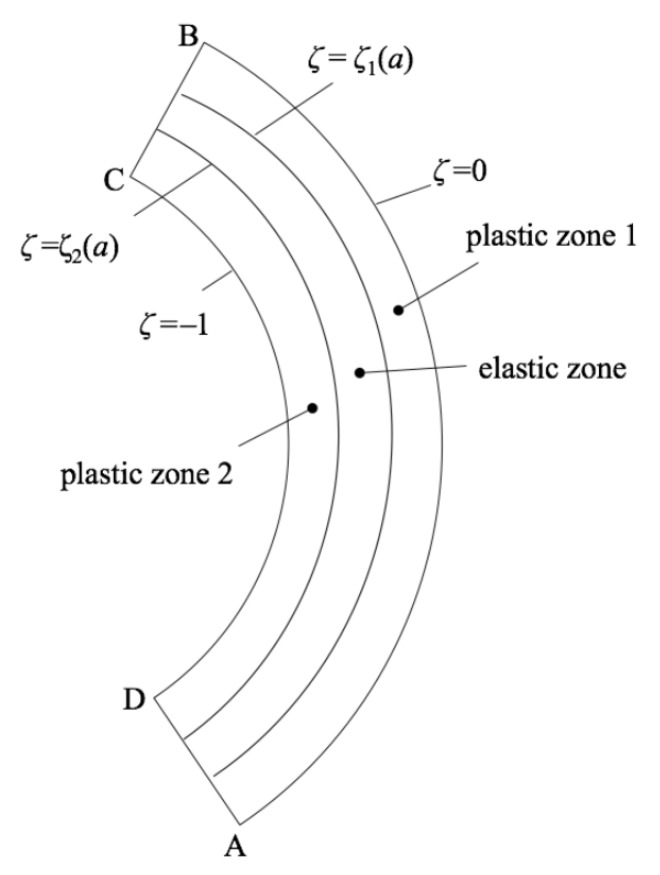
Schematics of elastic and plastic zones.

It is assumed that the functions β(ζ) and g(ζ) involved in Equations (7) and (9) are such that plastic yielding can only initiate at ζ=0 or ζ=−1. This assumption can be verified using the purely elastic solution with no difficulty. At the very beginning of the process, the entire sheet is elastic. As deformation proceeds, one of the following three cases arises: (i) plastic yielding initiates at the surface ζ=−1; (ii) plastic yielding initiates at the surface ζ=0; and (iii) plastic yielding initiates simultaneously at the surfaces ζ=−1 and ζ=0. These cases should be treated separately. In the following, $\zeta_{1}$ is the elastic/plastic boundary between the plastic region that propagates from the surface ζ=0 and the elastic region and $\zeta_{2}$ is the elastic/plastic boundary between the plastic region that propagates from the surface ζ=−1 and the elastic region. It is evident that both $\zeta_{1}$ and $\zeta_{2}$ depend on *a*. The general structure of the solution with two plastic regions is illustrated in Figure 2. Let *M* be the bending moment. Then, its dimensionless representation is in terms of the Lagrangian coordinates given by [18]

(11) $$\begin{array}{c} m=\frac{2\sqrt{3}M}{\sigma_{0}H^{2}}=\frac{\sqrt{3}}{a}\int_{-1}^{0}\frac{\sigma_{\eta }}{\sigma_{0}}d\zeta . \end{array}$$

In the elastic region, the whole strain is elastic. Therefore, it follows from Equation (1) that the principal logarithmic strains are

(12) $$\begin{array}{c} 2\varepsilon_{\zeta }^{e}=-2\varepsilon_{\eta }^{e}=-\ln\left[4\left(\zeta a+s\right)\right]. \end{array}$$

Since $\sigma_{\zeta }-\sigma_{\eta }=\tau_{\zeta }-\tau_{\eta }$, Equations (5) and (9) combine to give

(13) $$\begin{array}{c} \frac{\partial \sigma_{\zeta }}{\partial \zeta }+\frac{G_{0}ag\left(\zeta \right)}{\left(\zeta a+s\right)}\left(\varepsilon_{\zeta }^{e}-\varepsilon_{\eta }^{e}\right)=0. \end{array}$$

Eliminating the strain components in Equation (13) by means of Equation (12) results in

(14) $$\begin{array}{c} \frac{\partial \sigma_{\zeta }}{\partial \zeta }-\frac{G_{0}ag\left(\zeta \right)}{\left(\zeta a+s\right)}\ln\left[4\left(\zeta a+s\right)\right]=0. \end{array}$$

Integrating this equation with respect to ζ and using the boundary condition in Equation (4) at ζ=0 leads to

(15) $$\begin{array}{c} \frac{\sigma_{\zeta }}{\sigma_{0}}=\frac{a}{3k}\int_{0}^{\zeta }\frac{g\left(\chi \right)\ln\left[4\left(\chi a+s\right)\right]}{\left(\chi a+s\right)}d\chi ,\;\frac{\sigma_{\eta }}{\sigma_{0}}=\frac{\sigma_{\zeta }}{\sigma_{0}}+\frac{2}{3k}g\left(\zeta \right)\ln\left[4\left(\zeta a+s\right)\right], \end{array}$$

where $k=\sigma_{0}/\left(3G_{0}\right)$ and χ is a dummy variable of integration. The expression for $\sigma_{\eta }$ in Equation (15) has been derived using the identity $\sigma_{\eta }=\sigma_{\zeta }-\tau_{\zeta }+\tau_{\eta }$, and Equations (9) and (12). In the case of the purely elastic solution, Equation (15) must satisfy the boundary condition in Equation (4) at ζ=−1. Then, the equation for the function s(a) is

(16) $$\begin{array}{c} \int_{-1}^{0}\frac{g\left(\chi \right)\ln\left[4\left(\chi a+s\right)\right]}{\left(\chi a+s\right)}d\chi =0. \end{array}$$

Using Equation (15), in which *s* should be eliminated by means of the solution of Equation (16), and the yield criterion in Equation (8), it is possible to determine which of the three cases mentioned above occurs for given material properties. Simultaneously, the value of *a* at which plastic yielding initiates is determined. This value of *a* is denoted as $a_{e}$. In the following, it is assumed that $a\geq a_{e}$. It is now necessary to consider Cases (i), (ii) and (iii) separately.

Case (i). There are two regions. A plastic region occupies the domain $-1\leq \zeta \leq \zeta_{2}$ and an elastic region the domain $\zeta_{2}\leq \zeta \leq 0$. Equation (15) is valid in the elastic region. However, the function s(a) is not determined from Equation (16). It is reasonable to assume that $\sigma_{\eta }<\sigma_{\zeta }$ in the plastic region. Therefore, the yield criterion in Equation (7) becomes

(17) $$\begin{array}{c} \sigma_{\zeta }-\sigma_{\eta }=\frac{2}{\sqrt{3}}\sigma_{0}\beta \left(\zeta \right). \end{array}$$

Substituting Equation (17) into Equation (5) and integrating yields the dependence of the stress $\sigma_{\zeta }$ on ζ. Using Equation (17) again provides the dependence of the stress $\sigma_{\eta }$ on ζ. As a result,

(18) $$\begin{array}{c} \frac{\sigma_{\zeta }}{\sigma_{0}}=-\frac{a}{\sqrt{3}}\int_{-1}^{\zeta }\frac{\beta \left(\chi \right)}{\left(\chi a+s\right)}d\chi ,\;\frac{\sigma_{\eta }}{\sigma_{0}}=\frac{\sigma_{\zeta }}{\sigma_{0}}-\frac{2}{\sqrt{3}}\beta \left(\zeta \right). \end{array}$$

It is evident that this solution satisfies the boundary condition in Equation (4) at ζ=−1. Both $\sigma_{\zeta }$ and $\sigma_{\eta }$ should be continuous across $\zeta =\zeta_{2}$. Consequently, $\tau_{\zeta }$ is continuous across $\zeta =\zeta_{2}$. The stress $\tau_{\zeta }$ on the elastic side of the elastic/plastic boundary is determined from Equation (15) and on the plastic side from Equation (8). Then, the condition of continuity of $\tau_{\zeta }$ across the surface $\zeta =\zeta_{2}$ is represented as

(19) $$\begin{array}{c} g\left(\zeta_{2}\right)\ln\left[4\left(\zeta_{2}a+s\right)\right]=-\sqrt{3}k\beta \left(\zeta_{2}\right). \end{array}$$

Solving this equation for *s* yields

(20) $$\begin{array}{c} s=\frac{1}{4}\exp\left[-\frac{\sqrt{3}k\beta \left(\zeta_{2}\right)}{g\left(\zeta_{2}\right)}\right]-\zeta_{2}a. \end{array}$$

Using Equations (15) and (18), the condition of continuity of $\sigma_{\zeta }$ across the surface $\zeta =\zeta_{2}$ is represented as

(21) $$\begin{array}{c} \int_{0}^{\zeta_{2}}\frac{g\left(\chi \right)\ln\left[4\left(\chi a+s\right)\right]}{\left(\chi a+s\right)}d\chi =-\sqrt{3}k\int_{-1}^{\zeta_{2}}\frac{\beta \left(\chi \right)}{\left(\chi a+s\right)}d\chi . \end{array}$$

In this equation, *s* can be eliminated by means of Equation (20). The resulting equation should be solved numerically to find $\zeta_{2}$ as a function of *a*. Then, *s* as a function of *a* is readily found from Equation (20). The yield criterion should be checked in the elastic region using the solution in Equation (15). The calculation should be stopped when the yield condition is satisfied at one point of the elastic region. Denote the corresponding value of *a* as $a_{2}$.

In Case (i), Equation (11) becomes

(22) $$\begin{array}{c} m=\frac{\sqrt{3}}{a}\int_{-1}^{\zeta_{2}}\left(\frac{\sigma_{\eta }}{\sigma_{0}}\right)d\zeta +\frac{\sqrt{3}}{a}\int_{\zeta_{2}}^{0}\left(\frac{\sigma_{\eta }}{\sigma_{0}}\right)d\zeta . \end{array}$$

In the first integrand, $\sigma_{\eta }/\sigma_{0}$ should be eliminated by means of Equation (18) and in the second by means of Equation (15).

Case (ii). There are two regions. A plastic region occupies the domain $\zeta_{1}\leq \zeta \leq 0$ and an elastic region the domain $-1\leq \zeta \leq \zeta_{1}$. The elastic solution in Equation (15) satisfies the boundary condition in Equation (4) at ζ=0. Therefore, it is convenient to rewrite this solution as

(23) $$\begin{array}{c} \frac{\sigma_{\zeta }}{\sigma_{0}}=\frac{a}{3k}\int_{-1}^{\zeta }\frac{g\left(\chi \right)\ln\left[4\left(\chi a+s\right)\right]}{\left(\chi a+s\right)}d\chi ,\;\frac{\sigma_{\eta }}{\sigma_{0}}=\frac{\sigma_{\zeta }}{\sigma_{0}}+\frac{2}{3k}g\left(\zeta \right)\ln\left[4\left(\zeta a+s\right)\right]. \end{array}$$

The elastic solution in this form satisfies the boundary condition in Equation (4) at ζ=−1. It is reasonable to assume that $\sigma_{\eta }>\sigma_{\zeta }$ in the plastic region. Therefore, the yield criterion in Equation (7) becomes

(24) $$\begin{array}{c} \sigma_{\zeta }-\sigma_{\eta }=-\frac{2}{\sqrt{3}}\sigma_{0}\beta \left(\zeta \right). \end{array}$$

Substituting Equation (24) into Equation (5) and integrating yields the dependence of the stress $\sigma_{\zeta }$ on ζ. Using Equation (24) again provides the dependence of the stress $\sigma_{\eta }$ on ζ. As a result,

(25) $$\begin{array}{c} \frac{\sigma_{\zeta }}{\sigma_{0}}=\frac{a}{\sqrt{3}}\int_{0}^{\zeta }\frac{\beta \left(\chi \right)}{\left(\chi a+s\right)}d\chi ,\;\frac{\sigma_{\eta }}{\sigma_{0}}=\frac{\sigma_{\zeta }}{\sigma_{0}}+\frac{2}{\sqrt{3}}\beta \left(\zeta \right). \end{array}$$

It is evident that this solution satisfies the boundary condition in Equation (4) at ζ=0. Both $\sigma_{\zeta }$ and $\sigma_{\eta }$ should be continuous across $\zeta =\zeta_{1}$. Consequently, $\tau_{\zeta }$ is continuous across $\zeta =\zeta_{1}$. The stress $\tau_{\zeta }$ on the elastic side of the elastic/plastic boundary is determined from Equation (23) and on the plastic side from Equation (8). Then, the condition of continuity of $\tau_{\zeta }$ across the surface $\zeta =\zeta_{1}$ is represented as

(26) $$\begin{array}{c} g\left(\zeta_{1}\right)\ln\left[4\left(\zeta_{1}a+s\right)\right]=\sqrt{3}k\beta \left(\zeta_{1}\right). \end{array}$$

Solving this equation for *s* yields

(27) $$\begin{array}{c} s=\frac{1}{4}\exp\left[-\frac{\sqrt{3}k\beta \left(\zeta_{1}\right)}{g\left(\zeta_{1}\right)}\right]-\zeta_{1}a. \end{array}$$

Using Equations (23) and (25), the condition of continuity of $\sigma_{\zeta }$ across the surface $\zeta =\zeta_{1}$ is represented as

(28) $$\begin{array}{c} \int_{-1}^{\zeta_{1}}\frac{g\left(\chi \right)\ln\left[4\left(\chi a+s\right)\right]}{\left(\chi a+s\right)}d\chi =\sqrt{3}k\int_{0}^{\zeta_{1}}\frac{\beta \left(\chi \right)}{\left(\chi a+s\right)}d\chi . \end{array}$$

In this equation, *s* can be eliminated by means of Equation (27). The resulting equation should be solved numerically to find $\zeta_{1}$ as a function of *a*. Then, *s* as a function of *a* is readily found from Equation (27). The yield criterion should be checked in the elastic region using the solution in Equation (23). The calculation should be stopped when the yield condition is satisfied at one point of the elastic region. Denote the corresponding value of *a* as $a_{1}$.

In Case (ii), Equation (11) becomes

(29) $$\begin{array}{c} m=\frac{\sqrt{3}}{a}\int_{-1}^{\zeta_{1}}\left(\frac{\sigma_{\eta }}{\sigma_{0}}\right)d\zeta +\frac{\sqrt{3}}{a}\int_{\zeta_{1}}^{0}\left(\frac{\sigma_{\eta }}{\sigma_{0}}\right)d\zeta . \end{array}$$

In the first integrand, $\sigma_{\eta }/\sigma_{0}$ should be eliminated by means of Equation (23) and in the second by means of Equation (25).

Case (iii). In this case, there are two plastic regions, $-1\leq \zeta \leq \zeta_{2}$ and $\zeta_{1}\leq \zeta \leq 0$, and one elastic region, $\zeta_{1}\leq \zeta \leq \zeta_{2}$. At the beginning of this stage of the process, $a=a_{1}$ and $\zeta_{2}=-1$ or $a=a_{2}$ and $\zeta_{1}=0$. Let $\sigma_{n1}$ be the value of $\sigma_{\zeta }$ at $\zeta =\zeta_{1}$ and $\sigma_{n2}$ be the value of $\sigma_{\zeta }$ at $\zeta =\zeta_{2}$. Then, the elastic solution in Equation (15) can be rewritten as

(30) $$\begin{array}{c} \frac{\sigma_{\zeta }}{\sigma_{0}}=\frac{a}{3k}\int_{\zeta_{1}}^{\zeta }\frac{g\left(\chi \right)\ln\left[4\left(\chi a+s\right)\right]}{\left(\chi a+s\right)}d\chi +\frac{\sigma_{n1}}{\sigma_{0}},\;\frac{\sigma_{\eta }}{\sigma_{0}}=\frac{\sigma_{\zeta }}{\sigma_{0}}+\frac{2}{3k}g\left(\zeta \right)\ln\left[4\left(\zeta a+s\right)\right]. \end{array}$$

It follows from this solution that

(31) $$\begin{array}{c} \frac{\sigma_{n2}}{\sigma_{0}}=\frac{a}{3k}\int_{\zeta_{1}}^{\zeta_{2}}\frac{g\left(\chi \right)\ln\left[4\left(\chi a+s\right)\right]}{\left(\chi a+s\right)}d\chi +\frac{\sigma_{n1}}{\sigma_{0}}. \end{array}$$

The solution in Equation (18) is valid in the plastic region $-1\leq \zeta \leq \zeta_{2}$ and the solution in Equation (25) in the plastic region $\zeta_{1}\leq \zeta \leq 0$. Then,

(32) $$\begin{array}{c} \frac{\sigma_{n2}}{\sigma_{0}}=-\frac{a}{\sqrt{3}}\int_{-1}^{\zeta_{2}}\frac{\beta \left(\chi \right)}{\left(\chi a+s\right)}d\chi , \end{array}$$

and

(33) $$\begin{array}{c} \frac{\sigma_{n1}}{\sigma_{0}}=\frac{a}{\sqrt{3}}\int_{0}^{\zeta_{1}}\frac{\beta \left(\chi \right)}{\left(\chi a+s\right)}d\chi . \end{array}$$

Equations (20) and (27) are valid. Therefore,

(34) $$\begin{array}{c} \exp\left[\frac{\sqrt{3}k\beta \left(\zeta_{1}\right)}{g\left(\zeta_{1}\right)}\right]-4\zeta_{1}a=\exp\left[-\frac{\sqrt{3}k\beta \left(\zeta_{2}\right)}{g\left(\zeta_{2}\right)}\right]-4\zeta_{2}a, \end{array}$$

and

(35) $$\begin{array}{c} a=\frac{1}{4\left(\zeta_{2}-\zeta_{1}\right)}\left\{\exp\left[-\frac{\sqrt{3}k\beta \left(\zeta_{2}\right)}{g\left(\zeta_{2}\right)}\right]-\exp\left[\frac{\sqrt{3}k\beta \left(\zeta_{1}\right)}{g\left(\zeta_{1}\right)}\right]\right\}. \end{array}$$

Equations (31)–(33) combine to give

(36) $$\begin{array}{c} \int_{-1}^{\zeta_{2}}\frac{\beta \left(\chi \right)}{\left(\chi a+s\right)}d\chi +\frac{1}{\sqrt{3}k}\int_{\zeta_{1}}^{\zeta_{2}}\frac{g\left(\chi \right)\ln\left[4\left(\chi a+s\right)\right]}{\left(\chi a+s\right)}d\chi +\int_{0}^{\zeta_{1}}\frac{\beta \left(\chi \right)}{\left(\chi a+s\right)}d\chi =0. \end{array}$$

Eliminating in this equation *s* by means of Equation (20) or Equation (27) and then *a* by means of Equation (35) supplies the equation to find $\zeta_{1}$ as a function of $\zeta_{2}$ (or $\zeta_{2}$ as a function of $\zeta_{1}$). Then, *a* as a function of $\zeta_{1}$ (or $\zeta_{2}$) is found from Equation (35) and *s* as a function of $\zeta_{1}$ (or $\zeta_{2}$) from Equation (20) or (27). The distribution of the stresses is determined from Equation (30) with the use of Equations (32) and (33) in the elastic region, from Equation (18) in the region $-1\leq \zeta \leq \zeta_{2}$ and from Equation (25) in the region $\zeta_{1}\leq \zeta \leq 0$.

In Case (iii), Equation (11) becomes

(37) $$\begin{array}{c} m=\frac{\sqrt{3}}{a}\int_{-1}^{\zeta_{2}}\frac{\sigma_{\eta }}{\sigma_{0}}d\zeta +\frac{\sqrt{3}}{a}\int_{\zeta_{2}}^{\zeta_{1}}\frac{\sigma_{\eta }}{\sigma_{0}}d\zeta +\frac{\sqrt{3}}{a}\int_{\zeta_{1}}^{0}\frac{\sigma_{\eta }}{\sigma_{0}}d\zeta . \end{array}$$

In the first integrand, $\sigma_{\eta }/\sigma_{0}$ should be eliminated by means of Equation (18), in the second by means of Equation (30) and the third by means of Equation (25). As usual, it is necessary to verify that the yield criterion is not violated in the elastic region.

## 4. Unloading

It is assumed that unloading is purely elastic. This assumption should be verified a posteriori. At this stage of the process, the strains can be considered as infinitesimal. Let $a_{f}$ and $s_{f}$ be the values of *a* and *s*, respectively, at the end of loading. These values are known from the solution given in the previous section. Using Equation (10), the values of $r_{\mathrm{CD}}$ and $r_{\mathrm{AB}}$ at the end of loading, $r_{\mathrm{CD}}^{f}$ and $r_{\mathrm{AB}}^{f}$, are determined as

(38) $$\begin{array}{c} \frac{r_{\mathrm{CD}}^{f}}{H}=\sqrt{\frac{s_{f}}{a_{f}^{2}}-\frac{1}{a_{f}}}\equiv R_{f},\;\frac{r_{\mathrm{AB}}^{f}}{H}=\frac{\sqrt{s_{f}}}{a_{f}}=r_{f}. \end{array}$$

It is convenient to introduce a polar coordinate system (r,θ) with the origin at $x=-H\sqrt{s_{f}}/a_{f}$ and y=0 (point $O_{1}$ in Figure 1). The coordinate curves of this coordinate system coincide with the coordinate curves of the (ζ,η)-coordinate system. Therefore, $\sigma_{\zeta }=\sigma_{r}$ and $\sigma_{\eta }=\sigma_{\theta }$ where $\sigma_{r}$ and $\sigma_{\theta }$ are the normal stresses in the polar coordinate system. Moreover, $r=R_{f}H$ at ζ=0 and $r=r_{f}H$ at ζ=−1. The equilibrium equation for the increment of the stresses, $\Delta \sigma_{\zeta }$ and $\Delta \sigma_{\eta }$, in the polar coordinate system can be written as

(39) $$\begin{array}{c} \frac{\partial \left(\Delta \sigma_{\zeta }\right)}{\partial \rho }=\frac{\Delta \sigma_{\eta }-\Delta \sigma_{\zeta }}{\rho }, \end{array}$$

where ρ=r/H. Since $\sigma_{\zeta }=0$ at ζ=0 and ζ=−1 at any stage of the process, the increment of this stress should satisfy the conditions

(40) $$\begin{array}{c} \Delta \sigma_{\zeta }=0, \end{array}$$

for ζ=0 and ζ=−1.

The displacement components from the configuration corresponding to the end of loading in the polar coordinate system are supposed to be

(41) $$\begin{array}{c} u_{r}=H\left(\frac{U_{0}R_{f}^{2}}{\rho }-\frac{\rho V_{0}}{2}\right)\;\mathrm{and}\;u_{\theta }=H\rho \theta V_{0}, \end{array}$$

where $U_{0}$ and $V_{0}$ are dimensionless constants. Using Equation (41), the increment of the normal strains in the polar coordinate system is determined as

(42) $$\begin{array}{c} \Delta \varepsilon_{r}=-\frac{V_{0}}{2}-\frac{U_{0}R_{f}^{2}}{\rho^{2}},\;\Delta \varepsilon_{\theta }=\frac{V_{0}}{2}+\frac{U_{0}R_{f}^{2}}{\rho^{2}}. \end{array}$$

The increment of the deviatoric stresses is found from Equation (42) and the Hooke’s law (Equation (9)) where the stresses and strains should be replaced with the corresponding increments. Then,

(43) $$\begin{array}{c} \Delta \tau_{r}=-G_{0}g\left(\zeta \right)\left(V_{0}+2U_{0}\frac{R_{f}^{2}}{\rho^{2}}\right),\;\Delta \tau_{\theta }=G_{0}g\left(\zeta \right)\left(V_{0}+2U_{0}\frac{R_{f}^{2}}{\rho^{2}}\right). \end{array}$$

Using this solution, the right hand side of Equation (36) can be rewritten as

(44) $$\begin{array}{c} \frac{\Delta \sigma_{\eta }-\Delta \sigma_{\zeta }}{\rho }=\frac{\Delta \sigma_{\theta }-\Delta \sigma_{r}}{\rho }=\frac{\Delta \tau_{\theta }-\Delta \tau_{r}}{\rho }=2G_{0}g\left(\zeta \right)\left(V_{0}+2U_{0}\frac{R_{f}^{2}}{\rho^{2}}\right). \end{array}$$

The Lagrangian coordinate ζ at the end of loading is expressed in terms of ρ as [18]

(45) $$\begin{array}{c} \zeta =\frac{\left(\rho^{2}a_{f}-s_{f}\right)}{a_{f}}. \end{array}$$

Using this equation, it is possible to eliminate ζ in Equation (44). Then, substituting Equation (44) into Equation (39) and integrating gives

(46) $$\begin{array}{c} \frac{\Delta \sigma_{\zeta }}{\sigma_{0}}=\frac{2}{3k}\int_{r_{f}}^{\rho }\frac{g\left(\zeta \right)}{\chi }\left(V_{0}+2U_{0}\frac{R_{f}^{2}}{\chi^{2}}\right)d\chi . \end{array}$$

It is evident that this solution satisfies the boundary condition in Equation (40) at ζ=−1 (or $\rho =r_{f}$). The other boundary conditions in Equations (40) and (46) combine to yield

(47) $$\begin{array}{c} V_{0}\int_{r_{f}}^{R_{f}}\frac{g\left(\zeta \right)}{\rho }d\rho +2U_{0}R_{f}^{2}\int_{r_{f}}^{R_{f}}\frac{g\left(\zeta \right)}{\rho^{3}}d\rho =0. \end{array}$$

Solving this equation for $V_{0}$ results in

(48) $$\begin{array}{c} V_{0}=-2U_{0}R_{f}^{2}\int_{r_{f}}^{R_{f}}\frac{g\left(\zeta \right)}{\rho^{3}}d\rho {\left[\int_{r_{f}}^{R_{f}}\frac{g\left(\zeta \right)}{\rho }d\rho \right]}^{-1}. \end{array}$$

Using Equations (43) and (46), it is possible to represent the distribution of $\Delta \sigma_{\eta }$ as

(49) $$\begin{array}{c} \frac{\Delta \sigma_{\eta }}{\sigma_{0}}=\frac{\Delta \sigma_{\zeta }}{\sigma_{0}}-\frac{2\Delta \tau_{r}}{\sigma_{0}}=\frac{2}{3k}\int_{r_{f}}^{\rho }\frac{g\left(\zeta \right)}{\chi }\left(V_{0}+2U_{0}\frac{R_{f}^{2}}{\chi^{2}}\right)d\chi +\frac{2}{3k}g\left(\zeta \right)\left(V_{0}+2U_{0}\frac{R_{f}^{2}}{\rho^{2}}\right). \end{array}$$

The constant $V_{0}$ can be eliminated in Equations (46) and (49) by means of Equation (48). It is then obvious that both $\Delta \sigma_{\zeta }$ and $\Delta \sigma_{\eta }$ are proportional to $U_{0}$. The distribution of the residual stresses follows from Equations (46) and (49) in the form

(50) $$\begin{array}{c} \begin{array}{c} \frac{\sigma_{\zeta }^{res}}{\sigma_{0}}=\frac{\sigma_{\zeta }^{f}}{\sigma_{0}}+\frac{2}{3k}\int_{r_{f}}^{\rho }\frac{g\left(\zeta \right)}{\mu }\left(V_{0}+2U_{0}\frac{R_{f}^{2}}{\chi^{2}}\right)d\chi ,\\ \frac{\sigma_{\eta }^{res}}{\sigma_{0}}=\frac{\sigma_{\eta }^{f}}{\sigma_{0}}+\frac{2}{3k}\int_{r_{f}}^{\rho }\frac{g\left(\zeta \right)}{\chi }\left(V_{0}+2U_{0}\frac{R_{f}^{2}}{\chi^{2}}\right)d\chi +\frac{2}{3k}g\left(\zeta \right)\left(V_{0}+2U_{0}\frac{R_{f}^{2}}{\rho^{2}}\right). \end{array} \end{array}$$

As before, ζ should be eliminated by means of Equation (45) and $V_{0}$ by means of Equation (48). The constant $U_{0}$ remains to be found. To this end, it is necessary to use the condition that the bending moment vanishes at the end of unloading. Using Equations (11) and (45), this condition can be represented as

(51) $$\begin{array}{c} \int_{R_{f}}^{r_{f}}\left(\frac{\sigma_{\eta }^{res}}{\sigma_{0}}\right)\rho d\rho =0. \end{array}$$

This equation should be solved for $U_{0}$ numerically. Then, Equation (50) supplies the distribution of the residual stresses. To verify that the solution given in Section 4 is valid, this distribution should be substituted into the yield criterion in Equation (7) where $\sigma_{\zeta }$ and $\sigma_{\eta }$ should be replaced with $\sigma_{\zeta }^{res}$ and $\sigma_{\eta }^{res}$, respectively. The left-hand side of Equation (7) should be less than or equal to $\left(2/\sqrt{3}\right)\sigma_{0}\beta \left(\zeta \right)$ in the range −1≤ζ≤0.

## 5. Numerical Examples

Several numerical examples are presented in this section, based on the analytical solutions developed in the previous sections. Our chosen modulus gradient function is $g\left(\zeta \right)=1+\left(G_{1}/G_{0}-1\right){\left(-\zeta \right)}^{N}$, and yield stress gradient function $\beta \left(\zeta \right)=1+\left(\sigma_{1}/\sigma_{0}-1\right){\left(-\zeta \right)}^{N}$. The power law exponent *N* controls the functional distribution of material properties along the thickness coordinate ζ. The power law distributions in modulus and yield stress with the same *N* have been proposed in the literature [26,27]. The material parameters used in our numerical calculations are listed in Table 1.

### 5.1. Homogeneous Sheet under Bending

**Figure 3:**
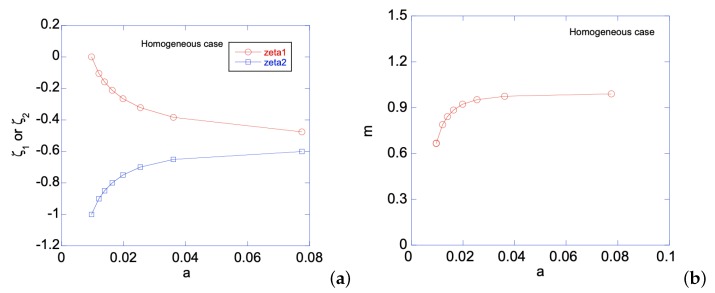
For the homogeneous sheet under bending: (**a**) $\zeta_{1}$ or $\zeta_{2}$ vs. *a*; and (**b**) applied bending moment *m* vs. *a*.

**Figure 4:**
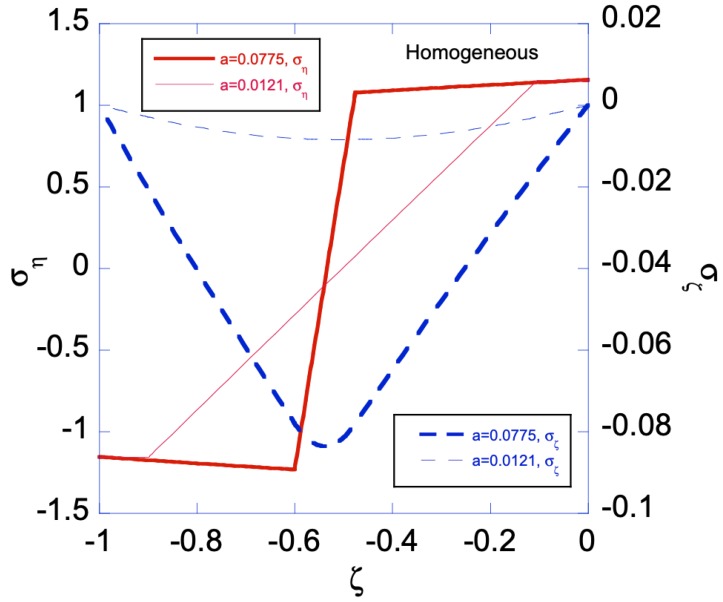
Stress distributions for the bent homogenous sheet with two different loading conditions $a=a_{1}^{h}=0.0121$ and $a=a_{2}^{h}=0.0775$. Two plastic zones, one developed from the left edge and the other from the right edge, increase their size as *a* increases for $\sigma_{\eta }$.

**Figure 5:**
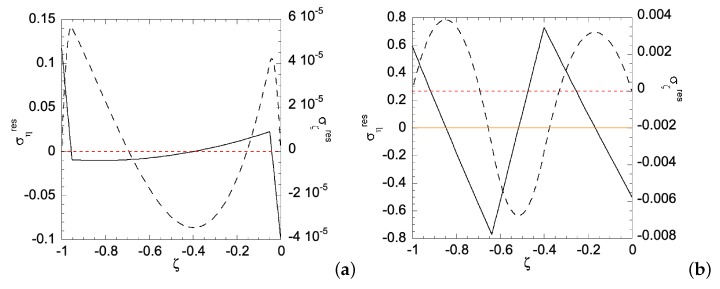
Residual stress distribution along the homogeneous sheet for: (**a**) $a=a_{r1}^{h}=0.0096$; and (**b**) $a=a_{r1}^{h}=0.0295$. Solid line is for residual $\sigma_{\eta }$ and dashed line for residual $\sigma_{\zeta }$. Zeros of $\sigma_{\eta }^{res}$ and $\sigma_{\zeta }^{res}$ are indicated by orange solid line and red dashed line, respectively.

When the homogenous sheet is under bending, both edges will simultaneously develop plastic zones. Figure 3a shows the movement of the two elastic-plastic boundaries toward the centerline of the sheet, as deformation magnitude increases. The deformation magnitude is measured by parameter *a*. The applied bending moment is a function of *a*, as shown in Figure 3b. As an illustration of the developed analytical solutions in previous sections, Figure 4 shows the stress distributions along the sheet under two different deformation magnitudes. As can be seen, the plastic zones increase with *a* for $\sigma_{\eta }$, while $\sigma_{\zeta }$ remains in elastic regime. The reason for $\sigma_{\eta }$ is not perfectly horizontal in the plastic zone is because our numerical codes do not allow *N* set equal to zero, hence a very small *N* is chosen, as shown in Table 1. After unloading, Figure 5a,b shows the residual stress distributions under two different *a*s. Larger *a* increases the magnitude of residual stresses after unloading. Moreover, the residual stress $\sigma_{\zeta }$ is zero at the left and right edges, as indicated by the red short-dashed line.

### 5.2. FGM Sheet Belonged to Case (i) under Bending

**Figure 6:**
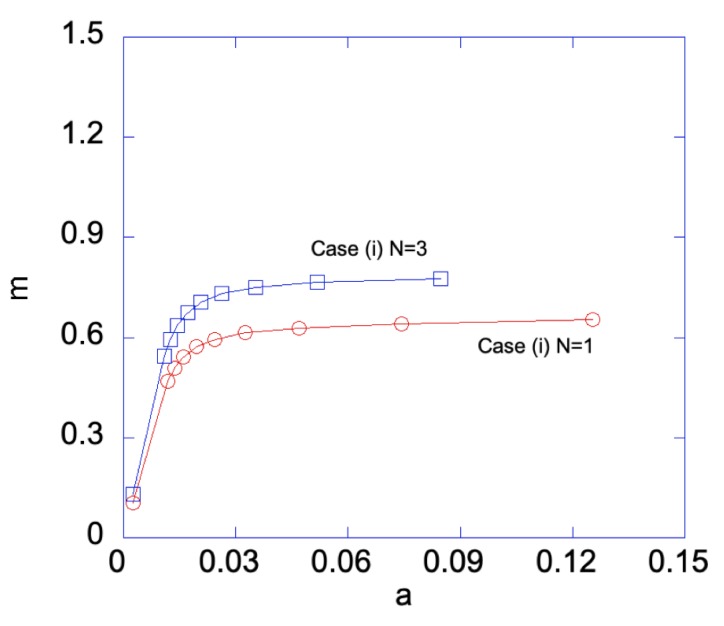
Bending moment *m* vs. *a* for Case (i) with N=1 and N=3. With sufficiently large deformation, i.e., large *a*, Case (iii) is automatically developed, hence both edges are plastically deformed.

**Figure 7:**
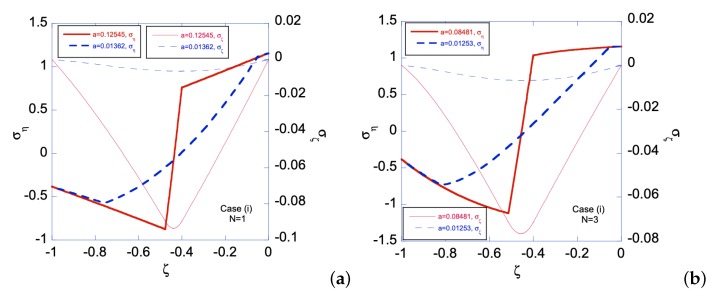
Stress distributions for Case (i) with *N* = 1 with two different deformation magnitudes, i.e., two different *a*s, for: (**a**) N=1; and (**b**) N=3. Two plastic zones, one developed from the left edge and the other from the right edge, increase their size as *a* increases for $\sigma_{\eta }$.

**Figure 8:**
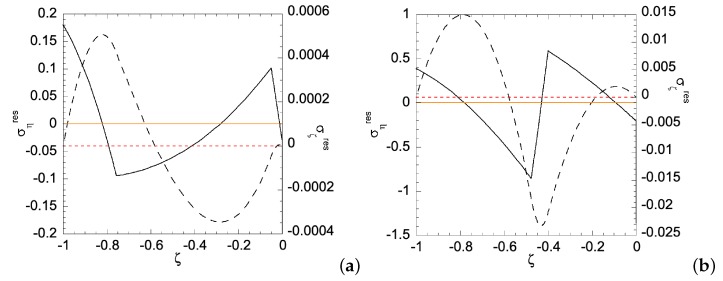
Residual stress distributions for Case (i) with *N* = 1 under deformation: (**a**) $a=a_{r1}^{\left(i\right)}=0.011785065$; and (**b**) $a=a_{r1}^{\left(i\right)}=0.074421129$. Solid line is for residual $\sigma_{\eta }$ and dashed line for residual $\sigma_{\zeta }$. Zeros of $\sigma_{\eta }^{res}$ and $\sigma_{\zeta }^{res}$ are indicated by orange solid line and red dashed line, respectively.

**Figure 9:**
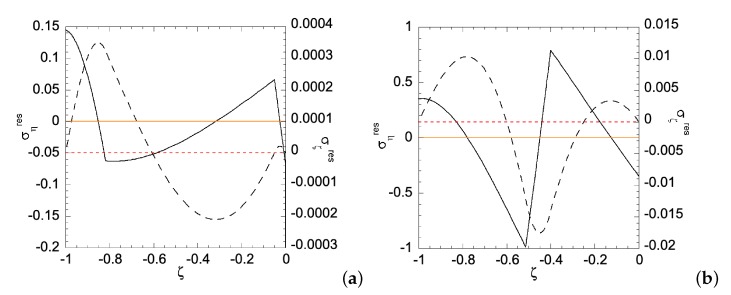
Residual stress distributions for Case (i) with *N* = 3 under deformation: (**a**) $a=a_{r3}^{\left(i\right)}=0.011023776$; and (**b**) $a=a_{r4}^{\left(i\right)}=0.051782217$. Solid line is for residual $\sigma_{\eta }$ and dashed line for residual $\sigma_{\zeta }$. Zeros of $\sigma_{\eta }^{res}$ and $\sigma_{\zeta }^{res}$ are indicated by orange solid line and red dashed line, respectively.

In Case (i), the left edge (ζ=−1) of the sheet has smaller yield stress, hence a plastic zone will start on the left edge first. Figure 6 shows the relationship between the applied bending moment and deformation magnitude a with the gradient function exponent *N* = 1 and 3. As can be seen, larger *m* is required for N=3 than that for N=1, as *a* increases. Under given deformation magnitudes, Figure 7 shows the stress distributions in the Case (i) FGM under plastic deformation. Larger plastic zone is developed at the left edge as deformation increases. After unloading, residual stress distributions are shown in Figure 8 and Figure 9 for the N=1 and N=3 FGM, respectively. Residual stresses are more predominant at the left edge. In addition, the residual stress $\sigma_{\zeta }$ is zero at the left and right edges, as indicated by the red short-dashed line.

### 5.3. FGM Sheet Belonged to Case (ii) under Bending

**Figure 10:**
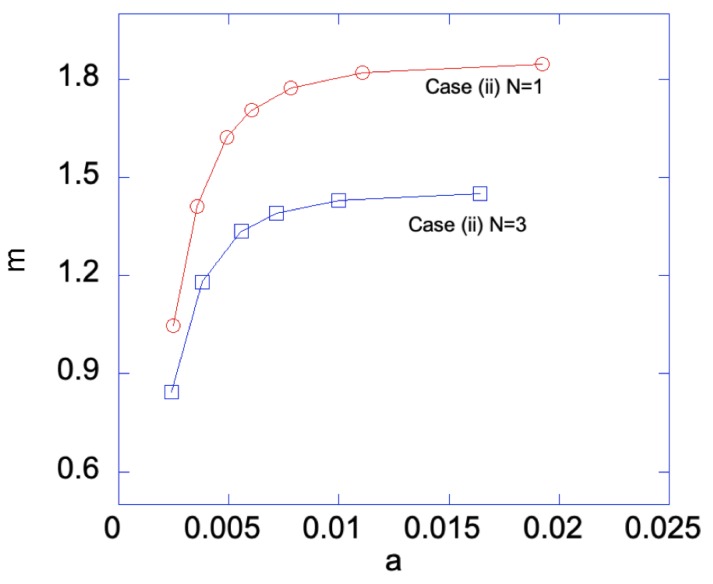
Bending moment *m* vs. *a* for Case (ii) with N=1 and N=3. With sufficiently large deformation, i.e., large *a*, Case (iii) is developed, hence both edges are plastically deformed.

**Figure 11:**
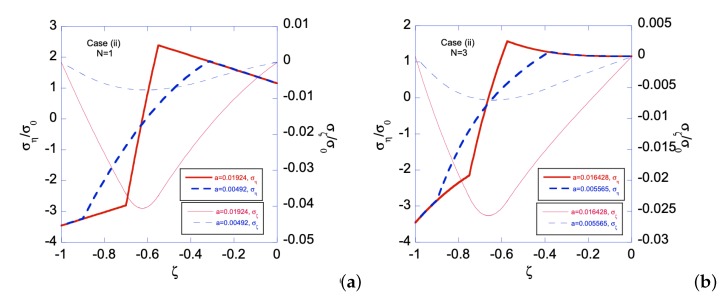
Stress distributions for Case (ii) with two different deformation magnitudes, i.e., two different *a*s, for: (**a**) N=1; and (**b**) N=3. Two plastic zones, one developed from the left edge and the other from the right edge, increase their size as *a* increases for $\sigma_{\eta }$.

**Figure 12:**
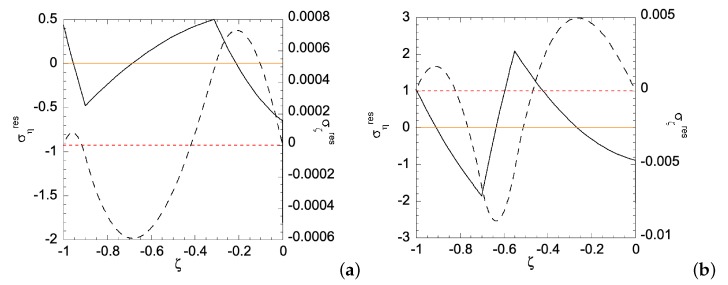
Residual stress distributions for Case (ii) with N=1 under deformation: (**a**) $a=a_{r1}^{\left(ii\right)}=0.004915193$; and (**b**) $a=a_{r1}^{\left(ii\right)}=0.019238382$. Solid curve is for residual $\sigma_{\eta }$ and dashed curve for residual $\sigma_{\zeta }$. Zeros of $\sigma_{\eta }^{res}$ and $\sigma_{\zeta }^{res}$ are indicated by orange solid line and red dashed line, respectively.

**Figure 13:**
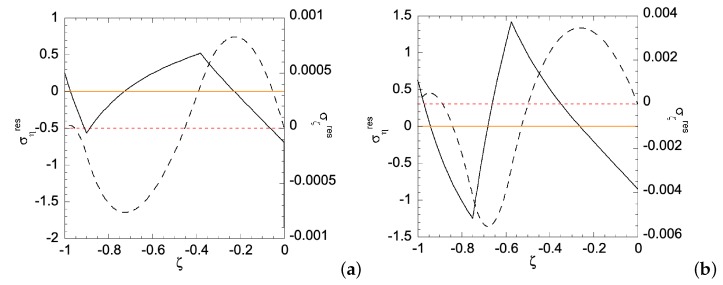
Residual stress distributions for Case (ii) with N=3 under deformation: (**a**) $a=a_{r3}^{\left(ii\right)}=0.005565273$; and (**b**) $a=a_{r4}^{\left(ii\right)}=0.016428139$. Solid curve is for residual $\sigma_{\eta }$ and dashed curve for residual $\sigma_{\zeta }$. Zeros of $\sigma_{\eta }^{res}$ and $\sigma_{\zeta }^{res}$ are indicated by orange solid line and red dashed line, respectively.

In Case (ii), the right edge (ζ=0) of the sheet has smaller yield stress, hence a plastic zone will start on the right edge first. Figure 10 shows the relationship between the applied bending moment and deformation magnitude a with the gradient function exponent *N* = 1 and 3. As can be seen, larger *m* is required for N=1 than that for N=3, as *a* increases. Under given deformation magnitudes, Figure 11 shows the stress distributions in the Case (ii) FGM under plastic deformation. Larger plastic zone is developed at the right edge as deformation increases. After unloading, residual stress distributions are shown in Figure 12 and Figure 13 for the N=1 and N=3 FGM, respectively. Residual stresses are more predominant at the right edge. The residual stress $\sigma_{\zeta }$ is zero at the left and right edges, as indicated by the red short-dashed line. The results from the illustrative examples solved here may serve as benchmark solutions for data obtained from numerical or experimental methods.

## 6. Conclusions

Efficient analytical and numerical methods and procedures have been developed and programmed to predict the distribution of stresses in a sheet of incompressible material subject to plane strain pure bending at large strain and then the distribution of residual stresses after unloading. Springback is also predicted. It has been assumed that the sheet is made of functionally graded material. The general theory has been developed for an arbitrary through thickness distribution of material properties assuming that the initiation of plastic yielding occurs at one of the surfaces of the sheet. This assumption can be verified using the purely elastic solution (Equation (15)) and the yield criterion (Equation (7)). It is possible to use the general solutions (Equations (15), (18) and (25)) even if the assumption is not satisfied but constants of integration should be added. Then, these general solutions should be combined to satisfy the boundary conditions and the conditions at elastic/plastic boundaries. An illustrative example is concentrated on power law distributions of material properties. Using the numerical code developed in this work enables the effect of parameters involved in these laws to be predicted effectively. The calculated examples show the analytical solutions derived here can systematically treat the plastic problems of the homogenous or functionally graded sheet. The magnitudes of applied moment may be strongly influenced by the power law exponent as deformation increases, which provides an effective way to design the functionally graded sheets.

The method employed to derive the solution in this paper can be extended to cyclic loading. This new solution may be useful for the interpretation of experimental data from the reverse bending fatigue test (for example, [28]).

## Figures and Tables

**Table 1:** Material parameters used in the numerical examples.

	$G_{0}$, GPa	$G_{1}$, GPa	$\sigma_{0}$, GPa	$\sigma_{1}$, GPa	*N*
Homogeneous	30	30	1	1	0.0001
FGM Case (i)	30	10	1	0.1	1
FGM Case (i)	30	10	1	0.1	3
FGM Case (ii)	10	30	0.1	1	1
FGM Case (ii)	10	30	0.1	1	3

## References

[1] Birman V., Byrd L.W. (2007). Modeling and analysis of functionally graded materials and structures. Appl. Mech. Rev..

[2] Tutuncu N., Ozturk M. (2001). Exact solutions for stresses in functionally graded pressure vessels. Compos. Part B Eng..

[3] Akis T. (2009). Elastoplastic analysis of functionally graded spherical pressure vessels. Comp. Mater. Sci..

[4] Sadeghian M., Toussi H.E. (2011). Axisymmetric yielding of functionally graded spherical vessel under thermos-mechanical loading. Comp. Mater. Sci..

[5] Li X.-Y., Li P.-D., Kang G.-Z. (2013). Axisymmetric thermo-elasticity field in a functionally graded circular plate of transversely isotropic material. Math. Mech. Solids.

[6] Horgan C.O., Chan A.M. (1999). The pressurized hollow cylinder or disk problem for functionally graded isotropic linearly elastic materials. J. Elast..

[7] Tutuncu N. (2007). Stresses in thick-walled FGM cylinders with exponentially-varying properties. Eng. Struct..

[8] You L.H., Wang J.X., Tang B.P. (2009). Deformations and stresses in annular disks made of functionally graded materials subjected to internal and/or external pressure. Meccanica.

[9] Jabbari M., Sohrabpour S., Eslami M.R. (2002). Mechanical and thermal stresses in a functionally graded hollow cylinder due to radially symmetric loads. Int. J. Press. Vessels Pip..

[10] Eraslan A.N., Akis T. (2006). Plane strain analytical solutions for a functionally graded elastic–plastic pressurized tube. Int. J. Press. Vessels Pip..

[11] Horgan C.O., Chan A.M. (1999). The stress response of functionally graded isotropic linearly elastic rotating disks. J. Elast..

[12] Peng X.-L., Li X.-F. (2012). Effects of gradient on stress distribution in rotating functionally graded solid disks. J. Mech. Sci. Technol..

[13] Bayat M., Saleem M., Sahari B.B., Hamouda A.M.S., Mahdi E. (2008). Analysis of functionally graded rotating disks with variable thickness. Mech. Res. Commun..

[14] Peng X.-L., Li X.-F. (2012). Elastic analysis of rotating functionally graded polar orthotropic disks. Int. J. Mech. Sci..

[15] Dai H.-L., Dai T., Zheng H.-Y. (2012). Stresses distributions in a rotating functionally graded piezoelectric hollow cylinder. Meccanica.

[16] Callioglu H., Sayer M., Demir E. (2015). Elastic–plastic stress analysis of rotating functionally graded discs. Thin-Walled Struct..

[17] Elishakoff I., Pentaras D., Gentilini C. (2016). Mechanics of Functionally Graded Material Structures.

[18] Alexandrov S., Kim J.-H., Chung K., Kang T.-J. (2006). An alternative approach to analysis of plane-strain pure bending at large strains. J. Strain Anal. Eng. Des..

[19] Hill R. (1950). The Mathematical Theory of Plasticity.

[20] Alexandrov S., Hwang Y.-M. (2009). The bending moment and springback in pure bending of anisotropic sheets. Int. J. Solids Struct..

[21] Alexandrov S., Hwang Y.-M. (2010). Plane strain bending with isotropic strain hardening at large strains. Trans. ASME J. Appl. Mech..

[22] Alexandrov S., Gelin J.-C. (2011). Plane strain pure bending of sheets with damage evolution at large strains. Int. J. Solids Struct..

[23] Alexandrov S., Hwang Y.-M. (2011). Influence of Bauschinger effect on springback and residual stresses in plane strain pure bending. Acta Mech..

[24] Verguts H., Sowerby R. (1975). The pure plastic bending of laminated sheet metals. Int. J. Mech. Sci..

[25] Romano G., Barretta R., Diaco M. (2014). Geometric continuum mechanics. Meccanica.

[26] Nakumura T., Wang T., Sampath S. (2000). Determination of properties of graded materials by inverse analysis and instrumented indentation. Acta Mater..

[27] Huang H., Chen B., Han Q. (2014). Investigation on buckling behaviors of elastoplastic functionally graded cylindrical shells subjected to torsional loads. Comput. Struct..

[28] Faghidian S.A., Jozie A., Sheykhloo M.J., Shamsi A. (2014). A novel method for analysis of fatigue life measurements based on modified Shepard method. Int. J. Fatigue.

